# Fixation method does not affect restoration of rotation center in hip replacements: A single-site retrospective study

**DOI:** 10.1186/1749-799X-7-25

**Published:** 2012-06-11

**Authors:** Alexander Wegner, Max Daniel Kauther, Stefan Landgraeber, Marius von Knoch

**Affiliations:** 1 Department of Trauma Surgery, University Hospital Essen, Essen, Germany; 2 Department of Orthopaedics, University Hospital Essen, Essen, Germany; 3 Department of Orthopaedics, Klinikum Bremerhaven Reinkenheide, Bremerhaven, Germany; 4 Department of Trauma Surgery, University Hospital Essen, Hufelandstr. 55, D-45147, Essen, Germany

**Keywords:** Hip replacement, BMI, Radiographic measurement

## Abstract

**Background:**

Aseptic loosening is one of the greatest problems in hip replacement surgery. The rotation center of the hip is believed to influence the longevity of fixation. The aim of this study was to compare the influence of cemented and cementless cup fixation techniques on the position of the center of rotation because cemented cup fixation requires the removal of more bone for solid fixation than the cementless technique.

**Methods:**

We retrospectively compared pre- and post-operative positions of the hip rotation center in 25 and 68 patients who underwent artificial hip replacements in our department in 2007 using cemented or cementless cup fixation, respectively, with digital radiographic image analysis.

**Results:**

The mean horizontal and vertical distances between the rotation center and the acetabular teardrop were compared in radiographic images taken pre- and post-operatively. The mean horizontal difference was −2.63 mm (range: -11.00 mm to 10.46 mm, standard deviation 4.23 mm) for patients who underwent cementless fixation, and −2.84 mm (range: -10.87 to 5.30 mm, standard deviation 4.59 mm) for patients who underwent cemented fixation. The mean vertical difference was 0.60 mm (range: -20.15 mm to 10.00 mm, standard deviation 3.93 mm) and 0.41 mm (range: -9.26 mm to 6.54 mm, standard deviation 3.58 mm) for the cementless and cemented fixation groups, respectively. The two fixation techniques had no significant difference on the position of the hip rotation center in the 93 patients in this study.

**Conclusions:**

The hip rotation center was similarly restored using either the cemented or cementless fixation techniques in this patient cohort, indicating that the fixation technique itself does not interfere with the position of the center of rotation. To completely answer this question further studies with more patients are needed.

## Introduction

Artificial hip replacement has become one of the standard procedures in orthopedic surgery, with a history of more than 100 years. Alone in Germany, 210.000 hip replacements were performed in 2010 [1]. World-wide, this number reached 800.000 hip prostheses with an increasing tendency in 2011 [2]. Early failure of primary hip replacement from causes, such as infection, fracture or implant failure, is rare [3]. Long-term failure of artificial hip replacement is usually a results of aseptic loosening [4]. Wear particles lead to a local inflammatory reaction with stimulation of osteoclasts that finally results in particle-induced osteolysis around the implant [5,6].

Authors have attributed the importance of the correct position of the implant along with durability of materials (e.g. ceramics or cross-linked polyethylene) as reasons for early aseptic loosening. An inclination of 45° ± 10° and anteversion of 15° ± 10° of the cup is described to be the optimal position to prevent dislocation [7,8]. In 1988, Yoder et al. documented that a superior, lateral position of the rotation center of the artificial cup leads to higher loosening rates than an anatomical position [9]. Callaghan et al. reported higher loosening rates with a varus position or high positioning of the prosthesis [10]. All patients included in these two studies had cemented hip prostheses. Both authors concluded that the correct position of cup and stem is of great importance for implant durability and survival. Sexton et al. showed that high acetabular component inclination, high femoral offset and lateralization of the hip center resulted in increased mechanical forces across the hip joint and shortened standing time [11]. Others have also reported that the position of the hip rotation center influences the range of motion, dislocation rate and loosening rate of artificial joints [12-14]. To reduce a false positioning of the components, pre-operative planning has become a standard before surgery [15].

The type of fixation of stem and cup also has an influence on implant durability. According to the Swedish hip arthoplasty register the cemented cup increases standing time compared to the cementless one, but the reverse is true for cementing the stem, where the cementless stem achieves a better standing time [16]. Many studies have focused on different fixation techniques. A cementless, implanted cup saves more of the bone stock for further revisional surgeries, but requires a better quality of bone compared with cemented cups [17]. Cementing requires exposing cancellous bone for cement penetration. In view of this, we speculated that the position of the rotation center of cups fixated with the cemented technique would be higher than cementless acetabularly fixated cups. This study retrospectively measured the post-operative position of the rotation center of the cup in cemented and cementless primary hip replacements in comparison to the anatomic rotation center of the hip.

## Materials and methods

### Patients

The study was approved by the local Ethics Committee on March 3, 2008 (#08-3621) and is in accordance with official guidelines in the Declaration of Helsinki from 1996. All 409 patients undergoing artificial hip replacement in our department in 2007 were included in the study. Data from all patients were available for analysis, and included pre- and post-operative radiographs. We excluded 316 cases because of prior hip surgery, total hip arthroplasty on the contralateral side or total hip arthroplasty for reasons other than primary osteoarthritis, including displaced femoral neck fracture, avascular hip necrosis, rheumatic disease, femoral head deformity, hip dysplasia and autoimmune arthritis. Thus, our study consisted of 93 patients in total, including 25 patients with cemented cups (Contemporary, Stryker, Duisburg, Germany) and 68 patients with cementless cups (Duraloc, DePuy, Kirkel-Limbach, Germany). The femoral head diameter was 28 mm. Palacos® R (Heraeus Medical, Wehrheim, Germany) was used as bone cement. A lateral approach to the hip was used in all cases. Two patient subgroups were defined with either optimal or suboptimal reconstruction of the hip rotation center after the surgery. The horizontal and vertical difference between the postoperative rotation center of the cup and the preoperative anatomical rotation center was ≤ 5 mm in the group with optimal reconstruction. The post-operative flexion was obtained from patient discharge letters.

### Radiographic measurements

**Figure 1:**
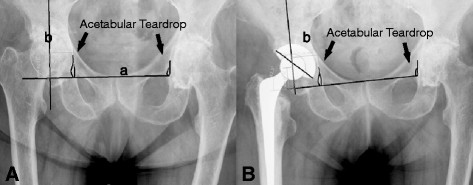
**Illustration of how the center of rotation prior to (A) and after (B) implantation of a cementless cup was determined when the acetabular teardrop was not visible.** Line a is drawn through the top of the foramina obtoratoria, and line b is drawn at a right angle to line a through the center of rotation. The vertical distance is measured from line a to the center of rotation, and the horizontal distance from the Köhler line (drawn along the medial aspect of the ilium and ischium) to the center of rotation.

**Figure 2:**
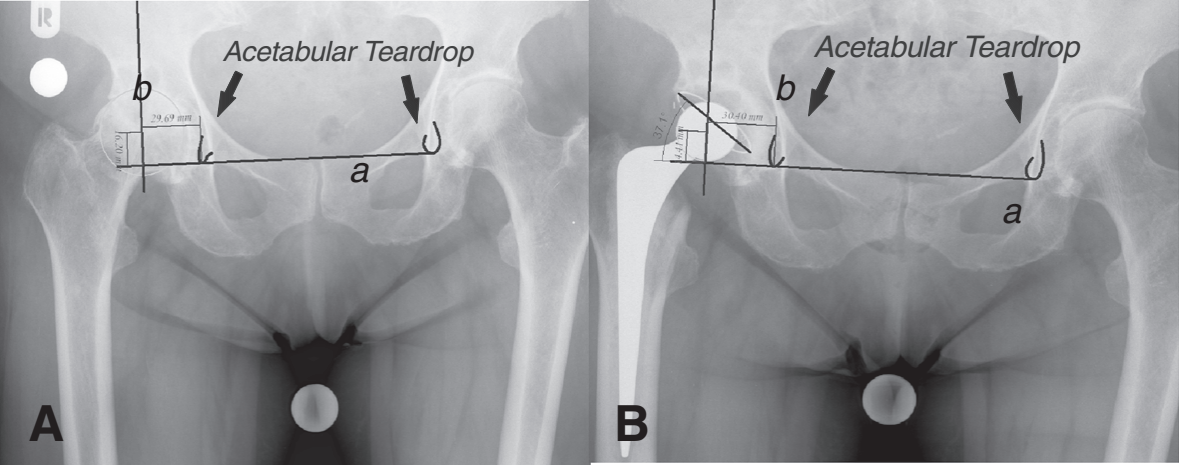
**Illustration of how the center of rotation prior to (A) and after (B) implantation of a cemented cup was determined when the acetabular teardrop was not visible.** Line a is drawn through the top of the foramina obtoratoria, and line b is drawn at a right angle to line a through the center of rotation. The vertical distance is measured from line a to the center of rotation, and the horizontal distance from the Köhler Line (drawn along the medial aspect of the ilium and ischium) to the center of rotation.

**Figure 3:**
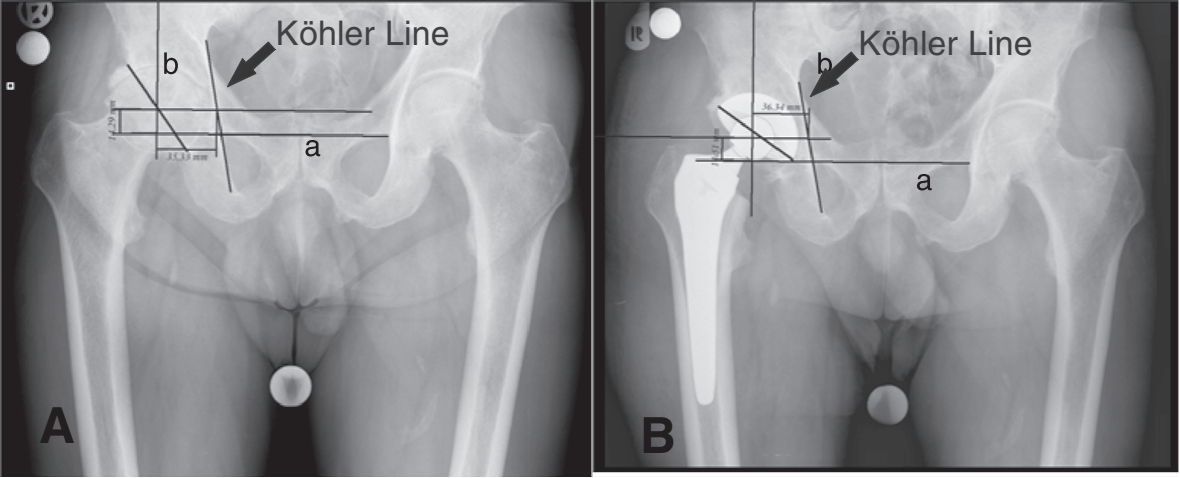
**Illustration of how the center of rotation prior to (A) and after (B) implantation of a cementless cup was determined.** Line a is drawn through the base of the acetabular teardrop, and line b is drawn at a right angle to line a through the center of rotation. The size guide is not included in the figure because it is only an illustration how the measurements were done.

**Figure 4:**
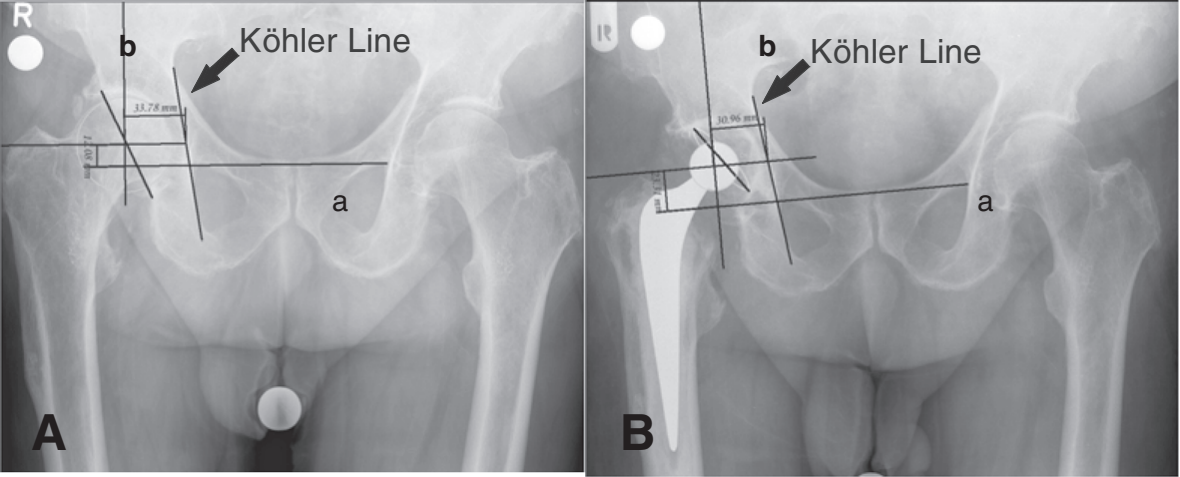
**Illustration of measurements determining the center of rotation prior to (A) and after (B) implantation of a cemented cup.** Line a is drawn through the base of the acetabular teardrop, and line b is drawn at a right angle to line a through the center of rotation.

We used pre-operative and 10-day post-operative digitalized anteroposterior x-rays of the pelvis centered over the pubic symphysis taken from a standard source to an object distance of 1 m. To evaluate the magnification of the radiographic images, we used a coin of known size (1.95 cm) on the radio film. The x-rays were all taken with the same equipment and by the same technicians. Two of the authors (AW and SL) independently measured the digital images using a digital image analysis system (MediCad II, HecTec., Niederviehbach, Germany). The mean values of both measurements were used in the analysis after the data were tested for significant differences. Measurements were started by drawing a line through the base of the acetabular teardrop, and determining three radiographic landmarks in the acetabulum: the teardrop base, the ilio-ischial line and the superolateral acetabular margin. We used the acetabular teardrop, first described by Köhler, as a reference because it has been described as an accurate method to measure distances [18,19]. The teardrop is located inferomedially in the acetabulum, just superior to the obturator foramen. Its lateral lip indicates the exterior and medial lip indicates the interior acetabular wall [20]. If the base of the teardrop was not clearly visible, we used a horizontal line drawn through the most proximal aspects of both foramina obturatoria and measured the distance between the drawn line and the center of rotation for the vertical distance. Horizontal distance was measured as the distance between the rotation center and the Köhlerline drawn along the medial aspect of the ilium and ischium [18] (Figure 1 and 2). The center of rotation was determined by drawing a circle around the femoral head [21]. The vertical and horizontal distances from the most distal point of the teardrop were measured. On the radiograph conducted 10 days post-surgery, we measured the horizontal and vertical distances of the artificial femoral head center from the most distal point of the teardrops (Figures 3 and 4).

### Statistics

We used the Kolmogorov-Smirnov test to assess the data distribution (normal: p < 0.05; non-normal: p ≥ 0.05). Differences between groups in normally distributed data were tested using the *t*-test and in non-normally distributed data using the Mann–Whitney *U* test. In all cases the two-sided tests were chosen. Correlations between multiple items were assessed using the Pearson correlation coefficient for normally distributed data and the Spearman correlation coefficient for non-normally distributed data. A p-value of 0.05 was considered as statistically significant. Power analysis was done according to the description of Cohen [22].

## Results

**Figure 5:**
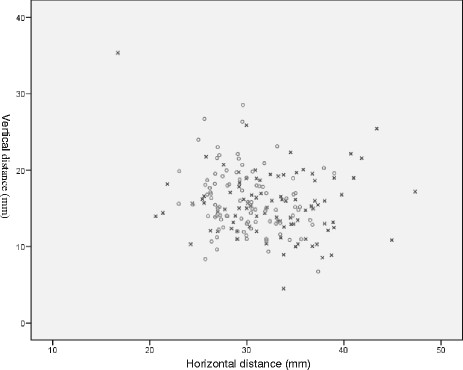
**Scatter-plot of the pre- and post-operative positions of the hip rotation center for all patients.** Pre- and post-operative positions are indicated by x and o in the plot, respectively. Distances were measured between the acetabular teardrop and center of hip rotation.

Mean patient age was 69 years (range: 33 to 95), and 41 and 52 prostheses were implanted on the left and right sides, respectively. The mean, minimum (min) and maximum (max) differences to the rotation center for patients in the cemented and cementless groups with standard deviations (SD) are summarized in Tables 1 and 2. The mean horizontal and vertical distances between the rotation center and acetabular teardrop were compared in pre- and post-operative radiographic images. The mean horizontal difference between pre- and post-operative images was −2.63 mm ± 4.23 mm (range: -11.00 mm to 10.46 mm) for the cementless and −2.84 mm ± 4.59 mm (range: -10.87 to 5.30 mm) for the cemented groups. The mean vertical difference between pre- and post-operative images was 0.60 mm ± 3.93 mm (range: -20.15 mm to 10.00 mm) for the cementless and 0.41 mm ± 3.58 mm (range: -9.26 mm to 6.54 mm) for the cemented groups. Based on our differentiation into two subgroups, we had 55 (59%) patients with optimal reconstruction and 38 (41%) patients with suboptimal reconstruction of the hip rotation center. From the 38 patients with suboptimal reconstruction, 9 (36% of all patients receiving cemented prostheses) received cemented fixation and 29 (43% of all patients receiving cementless prostheses) received cementless fixation. From the 55 patients with optimal reconstruction, 16 (64% of all cemented prostheses) received cemented fixation and 39 (57% of all cementless prostheses) received cementless fixation. Analysis of all of the data presented here shows no significant difference in the horizontal and vertical positioning of the hip rotation center before and after surgery for patients receiving either cemented or cementless fixation (Figure 5).

**Table 1:** Summary data for the 68 patients receiving cementless acetabular components.

	**Age**	**Vertical Distance**		**Vertical difference**	**Horizontal Distance**		**Horizontal difference**
		**Pre-op**	**Post-op**		**Pre-op**	**Post-op**	
Mean	66	15.22	15.83	0.60	32.39	29.75	−2.63
Minimum	33	5	7	−20	17	23	−11
Maximum	85	35	29	10	47	39	10
Standard deviation	9	4.44	4.36	3.93	5.23	3.33	4.23

distances in mm.

**Table 2:** Summary data for the 25 patients receiving cemented acetabular components.

	**Age**	**Vertical Distance**		**Vertical difference**	**Horizontal Distance**		**Horizontal difference**
		**Pre-op**	**Post-op**		**Pre-op**	**Post-op**	
Mean	78	16.27	16.69	0.41	33.15	30.30	−2.84
Minimum	60	9	11	−9	21	23	−11
Maximum	95	25	23	7	43	38	5
Standard deviation	7.37	3.97	3.30	3.58	5.70	3.97	4.59
P-value	**<0.001**	0.30	0.37	0.83	0.55	0.84	0.30

distances in mm; p-values determined using the *t*-test comparing the cemented and the cementless groups.

Measurements made indepentently by two residents did not differ significantly. There was no correlation between the position of the hip rotation center and the ability of flexion. All patients had good post-operative flexion (Table 3). Comparison of patients receiving cementless or cemented fixation revealed that post-operative flexion was significantly better in the group receiving cemented fixation (Table 4). A significant anti-proportional correlation between the vertical position of the hip rotation center and post-operative flexion (p < 0.05 r = −0.401, Table 5) was detected in patients classified as having optimal reconstruction of the anatomical hip rotation center. This correlation was not present in the group of patients defined as having suboptimal reconstruction of the hip rotation center. The horizontal position of the hip rotation center did not significantly correlate with post-operative flexion in either group, and “optimal” or “suboptimal” hip rotation center reconstruction also did not significantly correlate with post-operative flexion (Table 5, p = 0.335). After analysis of many factors that could also have contributed to the success of the hip replacement or our method of evaluation, we conlcude that the type of fixation did not influence the ability to restore the hip rotion center.

**Table 3:** Pre- and post-operative flexion in the entire patient cohort

	**Flexion**	
	**Pre-op**	**Post-op**
Mean	**87**	**86**
Minimum	**0**	**50**
Maximum	**120**	**105**
Standard deviation	**19**	**9**
p-Value	**0.111**	

**Table 4:** Flexion in patients receiving uncemented or cemented fixation

	**Patients with cementless fixation**		**Patients with cemented fixation**	
	**Pre-op Flexion**	**Post-op Flexion**	**Pre-op Flexion**	**Post-op Flexion**
Mean	**89**	**84**	**84**	**89**
Minimum	**0**	**50**	**10**	**70**
Maximum	**120**	**105**	**110**	**100**
Standard deviation	**18**	**10**	**20**	**6**
p-Value (cementless vs. cemented)	**Pre-op**		**Post-op**	
	**0.298**		**0.027**	

**Table 5:** Flexion of patients with optimal or suboptimal post-operative reconstruction of the hip rotation center

	**Patients with optimal reconstruction**		**Patients with suboptimal reconstruction**	
	**Pre-op Flexion**	**Post-op Flexion**	**Pre-op Flexion**	**Post-op Flexion**
Mean	**85.83**	**85.44**	**89.81**	**85.63**
Minimum	**0**	**70**	**50**	**50**
Maximum	**120**	**105**	**120**	**100**
Standard deviation	**21.53**	**7.62**	**12.77**	**11.55**
p-Value (pre- versus post-op Flexion)	**0.639**		**0.335**	

## Discussion

Several published reports on the preliminary results of total hip arthroplasty demonstrated that a superior and lateral position of the hip rotation center can lead to early aseptic loosening [9,10,23,24]. All of these reports, excepting the study from Pospula et al., observed patients with cemented prostheses. Lachiewicz et al. found that an acetabular cup placed 5 mm or more above the anatomical rotation center correlated with more frequent aseptic loosening. It has also been reported that a mispositioning of the hip rotation center can lead to a dislocation several years later [25]. Based on these reports, reconstruction of the exact anatomical position of the hip rotation center appears to be important.

The aim of our study was to assess the influence of fixation technique on the position of the hip rotation center. This study suggests that the technique used for fixation of the cup by itself does not necessarily influence the position of the hip rotation center. Both techniques can be used to reconstruct a physiological center of rotation (Figure 5). The difference between the cemented and cementless cup fixation techniques is that during the cementing procedure, the subchondral sclerotic zone must usually be partially penetrated or sometimes even removed to allow cement to penetrate into the bone for solid fixation. In view of this, we speculated that the rotation center of cups fixed with the cemented technique would be higher compared with cementless acetabular fixation. Interestingly, there was no significant difference in the position of the hip rotation center (Table 2). This is also supported by the close to equal distribution of patients with either fixation technique between the optimal and suboptimal reconstruction groups. These findings were confirmed in the study by Atilla et al., who reviewed patients with total hip arthroplasty reconstruction (cemented and cementless) with femoral bone autograft because of hip dysplasty. They observed that implant survival was significantly correlated with the position of the cup regardless of the fixation technique. A greater number of patients with a superolateral cup position experienced aseptic implant loosening [26]. We assume that the potential loss of the subchondral sclerotic zone was compensated for, at least in some cases, by the bone cement mantle which keeps the hip rotation center in the original position.

Pospula et al. observed a higher loosening rate if a cemented cup was used in comparison to cementless cup fixation [24]. Based on the results we present here, however, it is more likely that the position of the hip rotation center was the reason for the earlier loosening of cemented cups. Although, other reasons, such as errors within the cementing technique or greater bone loss during surgery, cannot be ruled out [27]. Osteonecrosis is another source that could affect cup loosening. The incidence of osteonecrosis is higher in patients receiving cup cementation because of the high temperatures required to set the cement and the leaking of toxic monomers from the cement [28]. Cement mantle fracture due to cement impurities or loss of strength of the cement over time could also be a reason for aseptic loosening [29]. The problem of increased Osteonecrosis could also be observed in other studies [30], although long-term results are better for cemented prostheses than for cementless prostheses [16]. The reason for this difference in outcome is unknown. It is possible that an unrecognized fracture occurring during surgery or enhanced wear after implantation of cementless prostheses could play a role. Patients receiving cementless prostheses have been reported to have a higher rate of revision because of fracture in the first year after surgery [16]. This could, at least in part, be because bone is more vulnerable after cementless fixation since it is not stabilized by the cement and because cementless prostheses are more often used in younger patients, who have a greater activity level [31]. It can be expected that wear will play a lesser role in patients operated on after 2000, since Bjerkholt et al. could show that the wear produced on new cementless prostheses is no longer different than for cemented ones [32].

If the hip rotation center is displaced by ≥ 1 cm from the anatomical position of the hip rotation center, the total hip failure rate is 6.44 times higher [26]. But none of the surgeries in our study resulted in a reconstructed hip rotation center ≥ 1 cm from the anatomical one (Tables 1 and 2). Nearly all surgeries in our series restored the hip rotation center more medial to the anatomical position. This often occurs as a result of losing floor osteophytes localized in the acetabulum near the pulvinar by normal reaming during the implantation procedure to reach the level of cortical bone and achieve good contact between the implant and bone [33].

The post-operative mean flexion in our patient cohort was 86°, which was nearly the same as pre-operative flexion (87°). Interestingly, flexion in the cemented group was significantly better than in the cementless group (table 4). This probably results from the higher vertical difference of the postoperative hip rotation center in the cementless group compared with the cemented group (0.6 vs. 0.41 mm), which also shortens the limb more in the cementless group, and is probably the root of the significant antipropotional correlation between the vertical hip rotation center and post-operative flexion in the group of patients with good reconstruction of the anatomical hip rotation center (p < 0.05 r = −0.401, Table 5). Since this correlation only occurred in patients with optimal, and not suboptimal, reconstruction, this could mean that limb length only affects post-operative flexion up to a point, at which it is no longer an effect. This correlation may also only be a random effect, since the power of this observation is limited by the fact that values are obtained during the hospital stay shortly after the surgery and the correlation coefficient is low (−0.405). Longer observation times could relate that differences are no longer significant later in recovery, but this situation was not considered in our study.

Our study has several limitations, the most important being the small number of patients which could be included. All exclusions were made because the original center of rotation could not be unequivocally determinated before surgery since its position was affected by underlying illness and its reconstruction would have made additional procedures necessary during surgery causing a bias for this patient group. The power of our study only reaches 0.15 with the small number of patients. To achieve a power of 0.6, 244 patients would need to be analyzed per group. Since data was generated only within one facility, it should be less influenced by institutional differences that are difficult to identify but, of course, cannot be extrapolated to all patients treated at all institutions. A total of six surgeons operated on the patients in the present study, but the influence of the surgeon could not be analyzed due to the small number of patients. It is conceivable, however, that one or more of the surgeons could have created a bias that clouded our results. Our study was also not randomized since it was carried out in retrospect, which limits its power. The influence of the position of the hip rotation center on prosthesis survival time or aseptic loosening was not assessed because sufficient follow-up data for this patient cohort was unavailable. Thus, we could not calculate the likelihood of aseptic loosening or other end points, such as fracture, infection or dislocation, for a non-anatomical position of the hip rotation center after arthroplasty. Because the coin used to normalize all measurments in x-rays for all patients on level of the film, it is possible that we created a bias in our measurements because patients with more tissue have a greater enlargement factor of their organs than thin patients (higher distance to film). Although BMI, age, hip flexion contracture, abductor muscle strength/weakness, surgical approach and bone density have also been reported to affect the hip rotation center, we did not analyze these factors here [34]. Patient age and bone density were not addressed in the study since we use cemented cups more often in elderly patients, thus, introducing a bias in the mean age that is a function of our descision process. The bone density should be poorer in the cemented group because of the higher age. Muscle strength was not directly analyzed, but we excluded all patients with a diagnosis other than primary osteoarthritis, and therefore, should not be a major factor within the patient group analyzed in our report. The surgical approach has not been considered because we only used the lateral approach to the hip.

The anteversion and inclination were not considered for two reasons. No effect on the position of the rotation center is expected from the middle of a spherical ball, which is the femoral head, and anteversion or inclination would only lead to a rotation around the rotation center and a higher rate of dislocation [7,8]. Even regarding the drawbacks of this single-site retrospective study, our data signal a necessity for further analyses of this type at other sites in order to improve the standing time and function of artificial hip joints and to answer the question which fixation method is the best for which patients.

We found no significant difference between the two fixation techniques on the position of the hip rotation center in patients treated at our center in 2007. The hip rotation center was similarly restored by both cemented and cementless fixation. Based on these results, we plan to maintain the strategy of using cementless fixation in young patients and cemented in older patients. While our results cannot be extrapolated to all patients receiving hip replacements in European or even German centers, it provokes the need for further assessment in more patients to increase the statistical power, and clarify this point for general recommendations for hip replacement surgery.

## **Competing interests**

The authors declare that they have no competing interests.

## **Authors’ contributions**

Conception and design of the study: AW, MvK; Analysis and interpretation of data: AW,MK, SL, MvK; Collection and assembly of data: AW, SL; Drafting of the article: AW, MK, SL, MvK. All authors read and approved the final manuscript.
